# Pill characterization data streams for reducing exposure to inadequately identified anti-malarial medication in developing countries

**DOI:** 10.1186/1475-2875-9-214

**Published:** 2010-07-22

**Authors:** Peter Pennefather, Aria Ilyad Ahmad, Ian Crandall, West Suhanic

**Affiliations:** 1 Laboratory for Collaborative Diagnostics, Leslie Dan Faculty of Pharmacy, University of Toronto, 144 College St, Toronto, M5 S 3M2, Canada

## Abstract

**Background:**

A large fraction of anti-malaria medicines (and indeed many other medicines classes) used in developing countries are inadequately identified. Framing this problem as one of misidentification rather than the more common framing of criminal misrepresentation leads to new solutions sets not currently being considered.

**Method:**

That reframing led to consideration and analysis of 4 new problems that informed design of a digital platform technology for delivering a distributed medicine characterization system: 1) problematic interests associated with a focus on preventing counterfeiting, 2) the complexity of the many ways that medicines can deviate from expected identities, 3) the challenge of choosing amongst a diversity of attribute characterization technologies, and 4) the need for a flexible and distributed data aggregation mechanism.

**Results:**

Analysis of those new problems confirmed an initial insight that a previously described digital technology for tracking malaria tests results in infrastructure limited regions could be adapted for characterizing pill attributes. Feasibility is illustrated by describing how the platform design can be implemented using open-source software and commodity computational and communication technology readily available and supportable in developing countries.

**Discussion:**

A system of this type would allow users to answer several questions. Is this medicine what it is supposed to be? Can it be used to treat locally encountered malaria? What has been the experience of others who have used pills having the same identity? Ubiquitous access to global digital telecommunication infrastructure allows the system to generate data streams from these distributed medicine characterization transactions that can be used to map global patterns of use of specifically identified medicines. This can provide feedback necessary to guide efforts to reduce the burden of malaria.

## Background

High-income countries have managed to reduce prevalence of inadequately identified medicines into their health systems to less than 1%. This achievement is the result of a strict regulatory framework governing the production and distribution of pharmaceutical products and harsh sanctions for offenders [1]. But, increased globalization of the market for medicine precursors and the medicines themselves has caused a proliferation of alternative formulations and is straining supply-side approaches to medicine use regulation [2,3].

Low- and middle-income countries face serious infrastructure limitations in the implementation of such supply-focused approaches to regulating medicines in use [4,5]. It estimated that as much as 25-50% of medicine purchased in developing countries deviate substantially from the pharmacopoeial specifications of the medicine that was intended to be purchased; a significant fraction are outright fakes [1,6-10]. Although direct characterization of anti-malaria medicines found in the health systems of several developing countries [6-8] have confirmed the seriousness of the problem, there is no mechanism for routinely characterizing the extent and dynamics of this misidentification problem in an on-going manner.

Low-income settings often demonstrate a relatively chaotic health care system [5] as well as resulting deficits in healthcare quality assessment and development capacity [4]. Information asymmetries [5] and endemic corruption [11] often subvert reliable but expensive supply-side quality assurance strategies developed for high-income country settings were expertise, resources, and infrastructure necessary for their implementation are readily available and costs can be rolled into the purchase price [9,11]. Although top-down reforms aimed at more policing and greater regulation of medicine producers and distributors are undoubtedly needed [1,6,9-11], this paper considers a complimentary alternative approach.

Framing the problem as one of misidentification rather than one of criminal misrepresentation of the pill suggests efforts should be put into developing a user-centred pill characterization system. Such a system would allow results of these material characterization transactions to be aggregated in real-time via digital networking technology, producing pill characterization data streams that can be shared over the Internet. This is important because exposures to medicines with mistaken identities represent a serious public health threat. Failure to realize what medications are actually being used results in a vicious cycle that is of particular relevance for understanding determinants of the global burden of infectious diseases like malaria. Infectious diseases in low-income settings are common. Treatment with anti-infective products that do not perform as expected because they are not what they are thought to be leads to a greater exposure to poorly treated disease than necessary thereby promoting development of drug resistance and further increasing disease burdens [8]. This situation also leads to a breakdown in trust in health system efforts to deal with that burden.

Implementation of a user-focused pill characterization system would improve clarity throughout the health system regarding the nature and distribution of anti-malarials in use. In order to improve effectiveness of drug therapy system-wide, it is necessary to communicate information about the nature of drugs being used to all participants in the system linking end-users to producers and dispensers. If the medication in question was deliberately produced to misrepresent its identity, greater access to information about the nature of the actual product being misrepresented will make it harder to get away with that deceit. If product identification was compromised inadvertently, the causes leading up to that mistake can be identified and dealt with. The methodology described here for tracking results of local medicine characterizations uses locally sustainable technology and easily supported web-based interpretive services. Such a system would enable a necessary feedback loop for guiding both local and system-wide treatment quality assessment efforts [9,10].

A user-focused pill characterization system must enable comparison of readily characterized identity metrics obtained with the product-at-hand to those associated with known medicine products (reference standards). Simple chemical analysis kits (based on for example thin layer chromatography) which enable direct comparison with reference standards have been used to provide rudimentary assessments of deviations from those reference standards. Such systems are currently being deployed as a means of evaluating the magnitude of the mistaken medicine problem [8] and as a means of extending the capacity of medicine regulatory authorities [12]. But, those simple analog test kits only provide a limited and local assessment of deviations [10]. This paper describes how a recently described tele-microbiology framework for distributed malaria diagnostic tests based on sharing telemicroscopy images of blood smears [13] can be adapted to help characterize identifying attributes of anti-malarial medicines. The design of this adaptation builds on recently described non-destructive digital spectroscopy technologies that can measure characteristic chemical signatures of pills even through packaging [14].

Digital identity metrics, coupled with ubiquitous digital information and communication infrastructure [15,16] enables creation of real-time medicine identification data streams that can be used to map global patterns of exposure to specific products sold as anti-malarials [3,17]. Real-time medicine identification data streams would begin to address one important aspect of existing information asymmetries commonly found in relatively unorganized health care markets. It would allow local users and distributors to know the answers to some important questions. Is this medicine-(product) what it is supposed to be? Can it be used to treat locally encountered malaria? What has been the experience of others who have used pills having the same identity? Answers to those questions would highlight which procurement policies deliver the most effective medicines possible within given sets of constraints (including price). Those answers would facilitate adaptation of medicine-use behaviour and future procurement strategies in ways that enhance end-user safety and generate more satisfactory medication experiences. At a minimum, distribution patterns for specific anti-malarial products and their imitators would become more evident. A combination of local experience and external support anchored by clarity around what anti-malarial products were actually used would then enable quality assessment systems to be developed and dangerous trends to be recognized early.

## Methods

Reframing the problem as medicine misidentification led to consideration and analysis of 4 new problems that informed design of a platform technology for delivering a distributed medicine characterization: 1) problematic interests associated with a focus on preventing counterfeiting, 2) the complexity of the many ways that medicines can deviate from expected identities, 3) the challenge of choosing amongst a diversity of attribute characterization technologies, and 4) the need for a flexible and distributed data aggregation mechanism.

### Problematic interests associated with a focus on preventing counterfeiting

Inadequate commercial incentives for making medicines affordable for the global majority [2,18] means that most people in the world today must either rely on charitable donations of medicines, with all the lack of sustainability that such reliance entails [19], or must seek out cheaper alternatives [20,21]. There is an unmet need for safe and effective medicines that is not readily satisfied by existing regulated sources. This unmet need encourages under-regulated sources [20] where there are many opportunities for misunderstanding and misrepresenting what is being acquired [3].

In developing countries, drug procurement can account for 40% of hospital expenditures [22]. This is a valuable resource that can encourage agents with access to the supply chain to divert supplies away from designated recipients or to infiltrate lower cost products and substitute these for higher cost products. Indeed, in many such countries large proportions (in some cases more than 50%) of procured medicines are reported to be "lost" during delivery [5,23]. What is lost is often found elsewhere. However, the quality and provenance of that diverted medicine is difficult to verify by end-users because of the circuitous route taken by such products to reach end-users.

Despite this lack of clarity as to provenance, semi-stable supply chains exist, with many individuals being aware of other agents upstream and downstream in these chains. These informal supply chains link multiple agents who simultaneously act as producers and consumers of goods and services within a complex external regulatory environment. Both licensed and informal agents deal with complex and often ineffective domestic regulatory regimes as well as with the diverse regulatory environments of trading partners supplying the medicines. These agents are constantly engaged in policy arbitrage where they scan the overall regulatory environment and determine how to best exploit it; the more chaotic this regulatory space, the greater the opportunity for personal gain at the expense of public good. The end-result is proliferation of supply chains where attempts at top-down regulation and standardization only create new policy arbitrage opportunities. Clarity around what was being supplied all along these informal supply chains enables self-policing behaviour to be reinforced while discouraging harmful acts such as deliberately making and selling fake medicines.

Global policy initiatives to address the specific threats of fake and substandard medicines exist [1,8], but developing a coordinated global response to this problem has proved challenging at best. The World Health Organization (WHO), for example, has enlisted the support of international organizations, enforcement agencies, national drug regulatory authorities, and NGOs to create the International Medicinal Products Anti-Counterfeiting Taskforce (IMPACT) through its 2006 Rome Declaration. Compliance with IMPACT initiatives however relies on voluntary international cooperation and national legislation harmonization. Controversies over guideline intentions have limited "IMPACT's impact" [3]. Also a recent economic analysis points out how from an industry perspective it is more profitable to frame the problem as one of abuse of intellectual property but from a public health perspective there is a better cost-benefit return by focusing instead on enabling local assessment of medicine effectiveness [23].

Thus, an overemphasis on the policing aspects of reducing exposure to counterfeit medicines is not only challenged by the voluntary nature of harmonization guidelines but also by a suspicions that the focus on property rights is more geared towards protecting market share of large pharmaceutical manufacturers rather than simply protecting public health. Instead of focusing regulations on meeting out punishment to purveyors of fake pills, a public health perspective aimed at determining identity of the medicines and adjusting therapy accordingly is suggested to prove more effective in the long-term at minimizing harm to users of anti-malarials.

### The complexity of the many ways that medicines can deviate from expected identities

**Figure 1:**
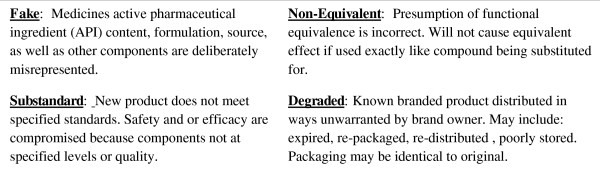
**Ways that medication identity can be mistaken**. The formulation and analytically determinable material attributes of the medicine-a- hand can deviate from that specified by pharmacopoeial monographs describing the medicine whose use was intended in several ways.

There are a variety of reasons that can lead to a presumed medicine being used as if it were a known product when it is in fact it is something else (Figure 1). In order to design a system that can help prevent such mistakes, it is important to distinguish between several subclasses of misidentified medicines. The term "fake" is used here instead of counterfeit to avoid conflating public health issues with intellectual property issues. Although "substandard" has been used to refer to all variants of a drug class that do not meet pharmacopoeial standards [9], it is more precise to restrict the term for products that result from manufacturing procedures that deviate from processes claimed by the manufacturers and distributors. The term "degraded" is used here to refer to changed identity occurring after manufacturing due to product instability. With known manufacturing standards and final product composition the expected rate and form of degradation can be predicted for specific locations and storage conditions.

"Non-equivalent" refers to a more subtle class of mistaken medicines. This is a catch-all class that includes all medicines that are substituted for another better known (and often branded) medicine but for a variety of reasons are not truly functionally equivalent (e.g. do not generate a biopharmaceutical response that closely matches, within specified limits, that of the other medicine being substituted for either deliberately or inadvertently). All of these different classes of misidentified medicines are thus different from non-conforming medicines which deviate from national formulary standards in a known way. Non-conforming medicines can be legally and ethically used if that known deviation is officially acknowledged and worked around.

Each of these types of deviations from the specified anti-malarial product identity have been detected in the field [6]. Indeed non-equivalent and substandard medicines seem more prevalent than outright fakes [8]. The prevalence of non-equivalent anti-malarial will only become more pronounced in the near future as the result of a conjunction of trends: 1) increasingly diversified generic market, 2) increasingly complex distribution chains, and 3) inevitable evolution of multiple forms of resistance to widely used anti-malarials.

A key first step in characterizing the utility of a medicine is identifying what it is. That knowledge in turns enables retrieval of information concerning best practices around use of that formulation. Recognition of the nature of a medicine's deviation from its presumed identity is needed to effectively determine whether the medicine can still be useful as a "non-conforming" medicine or should be simply be disposed of so that it will not be used inappropriately by others. Unfortunately non-conforming medicines often are all that is available at local distribution points and have often already been paid so it is important to explore whether they might still be useful. For example, the current system of routinely specifying 2-3 year expiry dates is driven primarily by marketing considerations. These products may still be effective and useful even though they are represented as degraded and therefore dangerous. Indeed, a recent direct test of the stability of expired fixed dose combination artemether-lumefantrine anti-malarial tablets in uncontrolled tropical conditions showed that chemical composition was stable as long as 5 years past the stated expiry date [22]. Under one system such expired products would have to be destroyed, under another a case could be made for their use. On the other hand, many compounds produced or procured as a specified formulation with specific bio-pharmaceutical properties do however, become poorer in quality over time because they are improperly stored. Yet, their external appearance and packaging may appear identical to the genuine article. Sharing experiences about effectiveness of accurately identified products with known provenance could inform users of risks associated with use.

### The challenge of choosing amongst a diversity of attribute characterization technologies

Any point-of-use assessment system must at a minimum allow end-users to verify the identity and source of the product they are using. End-users need to be able to directly confirm attributes characteristic of that product. In principle, packaging-based verification tools may be used [9,24] but these can still be subverted using modern print and packaging technology. Also, in many areas of the world, significant redistribution of pills occurs after the packaging has been removed [9,25]. More recent material focused variants of this tracking approach may be more difficult to subvert. For example, Laser Surface Authentication (LSA) takes advantage of the natural surface imperfections on product packaging or the pill itself to create an intrinsic signature that can be stored in an Internet accessible database or encrypted into a 2 D barcode on the packaging [26]. Low-cost portable scanners can then read that signal and verify authenticity of the product through comparison with a signature pattern registered with an international regulator or through local comparison with a known reference sample. Of course this approach can also be subverted but at a greater cost to the perpetrators.

Photonic methods can be adapted to analyse single tablets, for example by evaluating chemical taggants or coatings features [27]. Light-based analytical tools for verifying presence of active ingredients from spectral analysis of product composition are also becoming available and increasingly portable [14,26,27]. Some variants of Raman spectroscopy can be applied non-destructively through packaging at different points in the supply chain [14]. The chemically specific spectra generated also can be converted into unique digital identity codes for overt tagging. These approaches allow sequential quantitative testing of deviations from registered reference standards at different stages of the supply chain right up to the final dispensing step.

### The need for a flexible and distributed data aggregation mechanism

For such methods to be usefully incorporated into a data-feed there is a need for a distributed form of data aggregation and archiving. The Laboratory for Collaborative Diagnostics has developed a distributed means of recording large amounts of sequentially related image-based clinical laboratory diagnostic information. Use of this BioTIFF methodology in charactering globally distributed diagnostic testing has been described previously [13]. The same commodity cameras used in recording and sharing results of microscope based characterization of parasites can also be used to record and share spectral and image based analysis of pill attributes. Indexing these files and making the index network-accessible enables Google-like searches of the resulting database facilitating comparison of the attributes of the medicine-at-hand to reference standards. This would also allow medicine identity interrogators at all levels of the supply chain to compare their results with other recorded results and to form hypotheses about why the medicine has the signature properties that it has or on whether it is likely to be safe and effective if used as planned. A repository of medicine attribute characterization assessment results comprising of summaries of both measured spectra and other measured physical parameters as well as local knowledge of the medicine's origin could be built up by communicating local results to a data aggregation service using standard internet protocols or via telephony messaging modalities.

## Results and Discussion

### Real-time Medicine Characterization Data-Feeds

**Figure 2:**
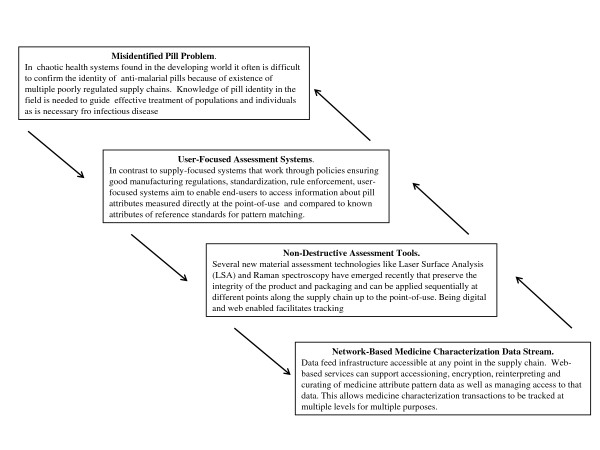
**Diagrammatic representation of key system design elements**. Four elements are specified: 1) the misidentified pill problem, 2) a user focussed assessment system, 3) non-destructive assessment tools, and 4) network-based medicine characterization data streams.

A system for tracking acquisition and intention to use specific medicinal products at a point-of-use emerges as a necessary element for ensuring safe and effective use of medicines in regions where national and local governments have limited capacity to regulate the types of medicines reaching end-users. Local assembly and implementation of a distributed user focused medicine characterization system seem feasible and possibly necessary [4]. Figure 2 presents a design schema for developing a network-based medicine characterization surveillance system that produces real-time data feeds of what is being encountered while serving a local need to know what medicine is being used.

The data feed produced by the proposed medicine attribute characterization system can simply be broadcast and tracked by interested parties or fed to network based aggregation services. The successful use of e-health kiosks and tele-centers in low-income settings [5] and the increasing portability and commoditization of the various forensic assessment methods like Raman spectroscopy and telemicroscopy [13,14,26,27] means that it is now possible, to deploy telemicroscopy-based medicine attribute assessment technology almost anywhere. For example, a cost effective spectrometer has been developed recently that is sensitive enough to discriminate between different brands of Tylenol locally available in Canada [28]. The open-source nature of much of the software needed to establish such networks means that there are no intellectual property barriers to developing a medicine attribute tracking network [13]. The use of non-destructive imaging also results in the use of fewer consumables. Costs-per-pill can be kept low by increasing throughput and amortizing up-front equipment costs over many tests. Most importantly however, the flexibility of being able to use any form of analytical device that can measure any useful identifying attribute of the pill allows the system to be easily adapted to local needs and contexts and supportable by local resources.

**Figure 3:**
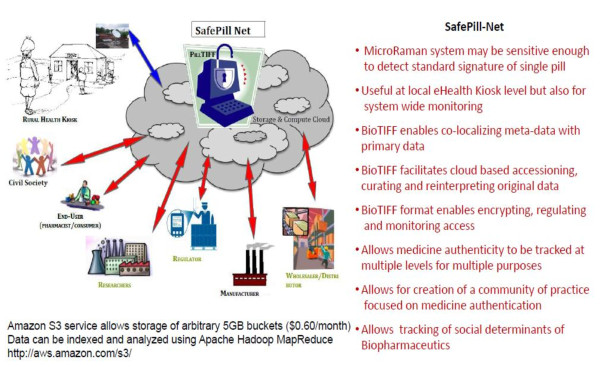
**Diagrammatic representation of stakeholders and expected results that could be associated with development of a safePill-net design**.

This paper describes a conceptual design rather than a fully operational system. Moreover, this design is such that it could be implemented in a variety of ways. Figure 3 illustrates the different stakeholders and settings that would have to be consulted and linked in order to set up such a pill characterization system and associated real-time data-feed. Recent attempts to objectively estimate the level of exposure to suspect medicines have focused on systematic random sampling methods [7-9]. In contrast, the design proposed is imagined first and foremost as a service to the medicine end-user. It aims to be locally controlled and sustained with the option of sharing results should that be deemed appropriate or necessary. In order to demonstrate the value of the design, it will be necessary to develop a training set of medicines to examine the capacity to deal with the diversity quality variations likely to be encountered in the field. This would entail requesting reference samples from registered global manufacturers and distributors of, for example, anti-malarial medications as well as sourcing samples of those medicines in use from an opportunistic global sample of diverse users. A multivariate principle component analysis using many analytical chemistry parameters combined with other determinants of medicine physical and chemical signatures could then be used to cluster commonly observed deviations from registered standards [29]. Prototyping the design will require collaboration with a suitable user group associated with a well defined health system.

Recently an advisory board convened by the manufacturer of Cortem^® ^(a fixed combination of atimisinin and lumefantrin) explored the problems and advantages of using that drug to treat asymptomatic malaria carriers [30]. It was pointed out that in many areas 30% of the population can be classified in that category and represents a reservoir that must be dealt with if the burden of disease is to be reduced. A system such as the one described above that combines confirmation of the medicine identity and a diagnostic designation, in a way that can be tracked and registered, could play an important role in ensuring success of such an approach. The use of such a system in post- marketing surveillance or in demonstrating utility of off-label applications of existing drugs by helping to register patient-observed-results may provide the necessary economic justification for private development of infrastructure enabling distributed identifying attribute characterization systems in high income countries.

## Conclusions

The major problem associated with exposure to medicines of uncertain identity in general and dubious anti-malarials in particular is that the degree to which their identity and utility is misrepresented is not easily verifiable by end-users. This state of affairs can arise for many reasons; the medicines can be fake, substandard, degraded, or non-equivalent imitations (see Figure 1). But the end-result is the same; the medicine is mistakenly believed to be a dosage form known to be safe and effective provided it is used as specified in a recognized pharmacopoeia. A combination of point-of-use analytics and mass-serialization now makes it feasible to evaluate drug provenance and identity (Figure 3) at each step in the medicine supply chain from production to final use. This approach allows end-users to verify the nature of products in their possession before use and adapt their procurement and use policies so as to maximize benefits.

The proposed system being digital and web enabled also allows for generation of real-time data feeds that can provide health system designers, participants and regulators with data about user experiences and expectation in the field. This information is essential for developing dynamic and adaptive medication monitoring systems [4]. While real-time data feeds cannot answer by themselves the question of whether the genuine article is effective under local conditions, it does allow that question to be asked without complications introduced by uncertainty as to what medicine was actually used. An opportunistic survey of the breadth of medicine types to which end-users in developing countries are being exposed to would help develop clearer hypotheses about causes and consequences of a pandemic of exposure to misiendentified medicines. Real-time medicine attribute characterization data streams will ensure that: 1) that the true life cycle of medications will be known, not guessed at; 2) the medication handed over at the point of sale will actually be known as meeting certain standards of therapeutic effectiveness or stability; and 3) unusual environmental conditions that affect medicine effectiveness can be monitored.

The system as described is designed simply to help end-users and their suppliers keep track of what is being used. In the future, as systems of this type become more widely deployed, manufacturers will have the opportunity to customize products for specific locations and needs and build web-based information services to replace printed package inserts explaining the purpose of the specific formulation design. It also allows for users to communicate their medication experience to manufacturers, regulators, and other users if they so chose. Also formulations may be specifically designed with excipients and coatings that make them more easily recognizable to digital analytical imaging devices as well as the human eye. This form of digital protective product packaging could support productive competition around costs and effectiveness. Innovative manufacturers will see the benefits of using this type of system to document and communicate reasons for believing their claims about why user should trust new lower cost innovative product designed for specific markets.

## Competing interests

PP, WS and IC are also principles in gDial Inc (www.gdial.ca) which is in the process of developing commercially sustainable "Bottom of the Pyramid" services based on some of the ideas presented in this paper. AA is M.Sc. candidate in the University of Toronto, Dept. of Pharmaceutical Science. He is co-supervised by PP and Jillian Kohler.

## Authors' contributions

PP wrote the initial draft of the manuscript. AA, IC, and WS contributed to critique of conceptual design and helped in revising multiple drafts. All authors read and approved the final manuscript

## Acknowledgements

Jillian Kohler, Warren Kaplan, and Paul Grootendorst are thanked for helpful comments.
